# Moderating effects of a healthy lifestyle on the association of pre-metabolic syndrome with multiple chronic disease comorbidities

**DOI:** 10.3389/fpubh.2025.1652015

**Published:** 2025-08-06

**Authors:** Youqiong Xu, Xinchao Zhang, Jinxi Fang, Wenchu Xu, Qihui Chen, Yitao Zhu, Haiping Hu, Xiangyu Cao, Xiaoyang Zhang

**Affiliations:** ^1^The Affiliated Fuzhou Center for Disease Control and Prevention of Fujian Medical University, Fuzhou, China; ^2^School of Public Health, Fujian Medical University, Fuzhou, China

**Keywords:** healthy lifestyle, pre-metabolic syndrome, comorbidities, risk factors, chronic disease healthy lifestyle

## Abstract

Studies have shown that healthy lifestyles reduce the risk of metabolic syndrome (MetS), but their impact on pre-metabolic syndrome (PreMetS) with multiple comorbidities remains unclear. To explore the association of PreMetS and MetS with multiple comorbidities and to assess whether a healthy lifestyle influences these associations. Associations between PreMetS and MetS, lifestyle behaviors and multiple comorbidities were analyzed by univariate and multivariate logistic regression. The moderating effect of healthy lifestyle was assessed by stratified analyses. Integrate healthy lifestyles and explore their association with multiple comorbidities using normal metabolism and healthy lifestyles as reference groups. PreMetS [OR = 1.38, 95%CI: 1.16–1.64] and MetS [OR = 1.61, 95%CI: 1.32–1.97] were associated with a significantly higher risk of multiple comorbidities compared with the normal population, and the risk of multiple comorbidities tended to increase as the number of metabolic disorder components increased (*p* < 0.001). Adherence to a healthy lifestyle (favorable [OR = 0.69, 95%CI: 0.59–0.82] and extremely favorable [OR = 0.54, 95%CI: 0.43–0.68]) was associated with a reduced risk of multiple comorbidities, with a trend toward a decreased risk of multiple comorbidities as the number of healthy lifestyles increased (*p* < 0.001). PreMetS was not associated with multiple comorbidities in healthy lifestyles (moderate and above) (*p* > 0.05), whereas MetS remained an associated risk factor for multiple comorbidities (*p* < 0.05). Compared to healthy lifestyle normometabolic subjects, unfavorable lifestyle PreMetS subjects were associated with increased risk of multiple comorbidities [OR = 2.05, 95%CI: 1.30–3.23], whereas healthy lifestyle PreMetS subjects were not associated with increased risk of multiple comorbidities [OR = 1.52, 95%CI: 0.93–2.50]. Metabolic profiles and lifestyle factors were independently associated with multiple comorbidities, and a healthy lifestyle counteracted the deleterious effects of PreMetS on the risk of multiple comorbidities in adults in Fuzhou. However, population homogeneity and recall bias resulting from the study design may lead to reverse causality and residual or unknown confounding factors.

## 1 Introduction

Metabolic syndrome (MetS) was introduced by Reaven in 1988 and is known as syndrome X (1). MetS is the most common form of obesity, dyslipidemia and hypertension. MetS is characterized by obesity, dyslipidemia, hyperglycemia and hypertension, and is now one of the major public health challenges worldwide (2). It is currently used as an early indication to recognize important adverse health outcomes in individuals, including cardiovascular disease (CVD) (3), cancer (4), chronic obstructive pulmonary disease (5), arthritis (6), spondylosis (7) and chronic kidney disease (8). Currently, the global prevalence of MetS ranges from 11.2% in low-income countries to 34.1% in upper-middle-income countries (9). As research has progressed, it has become clear that there is a phase known as pre-metabolic syndrome (PreMetS) that precedes the development of full-blown MetS. PreMetS is not a stand-alone disease, but rather a condition in which an individual exhibits cardiometabolic risk factors that fall short of the criteria for MetS (10, 11). Nonetheless, PreMetS increases the likelihood of MetS and can serve as an early warning sign for identifying cardiovascular health risks.

Recent studies have further revealed the pathophysiological mechanisms of PreMetS and MetS. Study (12) showed that pro-inflammatory imbalance is more pronounced in the progressive stage of PreMetS and precedes MetS, while the MetS stage is more characterized by a pronounced redox state imbalance. A 3-year follow-up study (13) found that inflammatory and oxidative stress markers were significantly increased in the MetS group compared to the non-MetS group. Further study (14) showed that leukocyte count, C-reactive protein, interleukin-6, and interleukin-10 concentrations were significantly higher in the PreMetS group compared to the normal group, whereas oxidative damage markers were significantly higher in the MetS group compared to PreMetS. The synergistic effects of PreMetS and MetS components and ongoing metabolic disorders greatly increase the mortality rate of the entire population and the risk of developing various diseases (15). Previous studies have shown that PreMetS patients are not only significantly associated with new-onset diabetes and hypertension (16) but also lead to an increased risk of gastrointestinal diseases (17) cardiovascular diseases, cancer (5) and dementia (18). The risk of CVD in MetS patients is 1.5–2.3 times higher than that in the normal population (15).

Lifestyle changes are considered to be a more effective non-pharmacological treatment and protection for patients with PreMetS and MetS. Studies have shown that a healthy diet (especially a vegetarian diet) (18, 19), cardiorespiratory endurance (20), or periodontal health (21) can have a beneficial effect on inflammation associated with PreMetS and MetS. In addition, non-smoking (22), moderate alcohol consumption (23), moderate-intensity exercise (24), high-quality diet (25), and appropriate sleep duration (26) were associated with a lower risk of developing MetS. Further study (27) has shown that a healthy lifestyle can reduce the risk of all-cause mortality associated with PreMetS and MetS, and this protective effect is more pronounced in older populations (<65 years of age). However, it remains unclear whether it can reduce the risk of multiple comorbidities. Therefore, there is a need for early identification of patients with PreMetS and MetS to guide further lifestyle interventions and anti-inflammatory treatments (28) to reduce the public health burden.

Lifestyle interventions are particularly important in the face of pathological changes at different stages. Healthy lifestyle interventions such as optimizing diet, increasing physical activity, controlling body weight, and quitting smoking and alcohol can help patients improve their metabolic disorders and reduce the risk of associated comorbidities. Based on the above, this study is the first to explore the association between lifestyle, PreMetS, and MetS with the risk of multiple comorbidities, which is essential to slow or prevent the number of comorbidities and to develop effective management and intervention strategies for previously screened individuals.

## 2 Populations and methods

### 2.1 Study populations

Using the PPS sampling method proportional to the population size, from June 2019 to June 2022, the 12 districts and counties of Fuzhou City were divided into three strata (urban areas, small and medium-sized cities, and rural areas), and a total of 30 towns (streets), 90 administrative villages (rural residents’ committees), and 180 residents’ groups, with at least 50 households in each residents’ group, and the KISH table was drawn to the respondents, and a total of ≥18 years of age permanent resident were surveyed a total of 9,155 residents were surveyed for face-to-face questionnaires, physical examinations and laboratory tests. A total of 158 people with missing MetS components were excluded, and a total of 8,997 study participants were included. A total of 60 people were further excluded from the six-item lifestyle questionnaire, and a total of 8,937 study participants were included. The study was approved by the Ethics Committee of Fuzhou Center for Disease Control and Prevention (approval number: 2022002). The procedures used in this study adhere to the tenets of the Declaration of Helsinki.

#### 2.1.1 Inclusion and exclusion criteria for the study populations

Inclusion criteria: permanent residents ≥18 years of age (≥180 days of residence in the area) with autonomy to participate in the survey and sign the informed consent form were selected.

Exclusion criteria: primary kidney disease, leukemia and malignancy, pernicious anemia, severe heart failure, endocrine system diseases and other recent major infections; pregnant and lactating women were excluded; and did not sign the informed consent form.

### 2.2 Content of the survey

#### 2.2.1 Questionnaire survey

The survey was conducted using a paper questionnaire uniformly designed by the Fuzhou Center for Disease Prevention and Control, which mainly included basic information: name, gender, date of birth, ethnicity, home address, marital status, education level, etc.; lifestyle: participation in smoking, alcohol consumption, physical exercise, sleep, dietary status.

#### 2.2.2 Physical examination

A uniformly equipped and calibrated height and weight scale, waist circumference (WC) ruler, and electronic blood pressure monitor (Omron HBP-1300 model) were used to measure the surveyed population. Height: The person being measured stood barefoot, in the “upright” position, on the base plate of the stadiometer, with the heel, sacral area and between the two shoulder blades resting firmly on the stadiometer’s column. The measurer stands both to the right and left of the person being measured, adjusts his/her head so that the upper edge of the ear screen is flush with the lowest point of the lower edge of the eye sockets, and then moves the horizontal plate of the height gage to the top of the head of the person being measured, so as to make it appropriately loose and tight. Weight: When measuring weight, remove shoes, hats and outerwear, stand firmly in the center of the scale, read the number after stabilization, take the number in kilograms and retain one decimal place. WC: Waist circumference was measured at the midpoint between the iliac crest and the lowest rib margin with a non-stretchable tape, while standing with feet 25–30 cm apart. Two measurements were taken to the nearest 0.1 cm and averaged. Blood pressure: Rest quietly for 5–10 min before measuring blood pressure. Measurement should be done with the upper arm outstretched and the sphygmomanometer, heart and cuff at the same level. Measure three times and take the average value.

#### 2.2.3 Laboratory tests

Including glucose tolerance test and blood biochemistry test, applying automatic biochemistry analyzer (Model 7020, Hitachi, Japan) and its supporting reagent kit for testing. Glucose tolerance test: including the detection of fasting blood glucose (FBG) level and 2-h postprandial blood glucose (OGTT-2 h). FBG was measured by collecting 3 mL of venous blood after 8–12 h of fasting. 75 g of dextrose was dissolved in 300 mL of warm water and consumed within 5 min and venous blood was collected after 2 h. Measurement of OGTT-2 h, i.e., 2 h after the glucose tolerance test. Biochemical tests: Serum total cholesterol (TC), Triglyceride (TG), low density lipoprotein-cholesterol (LDL-C), HDL-C, serum uric acid (SUA), blood urea nitrogen (BUN) and blood creatinine (Cre) levels. Serum TC was measured by enzyme colourimetric method, serum LDL-C, HDL-C and TG, SUA by colourimetric method, and serum FPG by hexokinase method (both Hitachi automatic biochemistry 7100). All laboratory tests were done by center for disease control and prevention in each district (county).

### 2.3 Definition of variants

#### 2.3.1 MetS and PreMetS

We used the ATP III criteria (2005) as revised by the National Cholesterol Education Program Adult Treatment Panel III (NCEP/ATP III) (29, 30), compared with the IDF standard and the JCDCG standard, this standard has the highest age-standardized prevalence rate (Supplementary Table S1). The diagnosis was made if three or more of the following were met: (I) abdominal obesity: ≥ 90 cm in men and ≥ 85 cm in women (based on population-and country/region-specific definitions); (II) high TG: TG ≥ 1.7 mmol/L, or those who have received treatment; (III) Reduced HDL-C: < 1.03 mmol/L for men and < 1.29 mmol/L for women, or those who have received treatment; (IV) Elevated blood pressure: SBP ≥ 130 mmHg or DBP ≥ 85 mmHg, or those who have a diagnosis of hypertension; (V) Elevated FPG: FPG ≥ 5.6 mmol/L or those who have a diagnosis of diabetes mellitus. PreMetS was defined as meeting one or two criteria for MetS.

#### 2.3.2 Smoking

Never having smoked was categorized as non-smoking (31).

#### 2.3.3 No or moderate alcohol consumption

We collected alcohol consumption, including red and white wine, beer and liquor. We multiplied the alcohol content (in grams) of a given portion by the frequency (days) and summed all alcoholic beverages to estimate the average alcohol consumption (g/day). Moderate alcohol consumption was defined as moderate drinking (women: 5–15 g/day; men: 5–30 g/day) (31).

#### 2.3.4 Healthy diet

The food frequency questionnaire collected information on intake of various food groups over the past year. Weekly intake of 6 food groups included daily intake of fresh fruit, fresh vegetables and milk, fish and other seafood ≥ 1 day per week, pulses and legumes ≥ 4 days per week and red meat < 7 days per week. For each food group, participants who met the criteria were awarded 1 point, otherwise 0 points (32, 33).

#### 2.3.5 Obesity/central obesity

BMI is a measure of overall obesity. According to the data from the physical examination of the study participants, BMI = weight/height^2^ (kg/m^2^). According to our standards, BMI < 18.5 kg/m^2^ was considered underweight; 18.5 kg/m^2^ ~ 24 kg/m^2^ was considered normal weight; 24 kg/m^2^ ~ 28 kg/m^2^ was considered overweight; and BMI ≥ 28 kg/m^2^ was considered obese; a healthy body weight was defined as 18.5 kg/m^2^ ~ 24.0 kg/m^2^. Central obesity was defined as a WC ≥ 90 cm for men and ≥ 85 cm for women. Central obesity was defined as WC ≥ 90 cm for men and ≥ 85 cm for women.

#### 2.3.6 Healthy sleep

Adequate sleep duration (7–8 h/day) was defined as healthy sleep (34, 35).

#### 2.3.7 Moderate-intensity exercise

Regular physical activity was defined as at least 150 min of moderate-intensity activity per week or 75 min of vigorous activity per week (or equivalent combination) (31).

In the current study, we considered 6 modifiable behavioral factors including smoking, alcohol consumption, body weight, physical activity, diet, and sleep duration and generated lifestyle scores. Of these, never having smoked, not drinking alcohol/moderate alcohol consumption, moderate to high intensity physical activity, healthy diet, and appropriate sleep duration were categorized as low risk. For each factor, a score of 1 was assigned to the low-risk level and 0 otherwise. Lifestyle scores were constructed from the sum of all 6 factors, ranging from 0 to 6, with higher scores indicating better adherence to an overall healthy lifestyle. To avoid a limited number of extreme groups of cases, the lifestyle score was subsequently divided into four groups: unfavorable (0–2), moderate (3), favorable (4), and extremely favorable (5–6) (27).

Multiple comorbidities in this study included the answer to the question, ‘Have you been diagnosed with any of the following diseases by a township health center or a community health service center or above: coronary heart disease/stroke/malignant neoplasm/chronic obstructive pulmonary disease/neck and low back disease/bone and joint disease/chronic urinary system disease?’ Participants who answered ‘yes’ to at least two of the seven questions were defined as having no or a single comorbidity for the rest of the participants.

### 2.4 Statistical analyses

R 4.2.2 software was used for statistical analysis. Normal information was expressed as mean ± standard deviation ( $\bar{x}$ *± s*) for measurement data, and comparisons of differences between groups were analyzed by ANOVA, and non-normal information was expressed as median M (upper quartile, lower quartile) (P_25_, P_75_), and comparisons of differences between groups were analyzed by non-parametric tests. Count data were expressed as frequency and constitutive ratio n (%), and comparisons of differences between groups were analyzed using the chi-square test.

Logistic regression analysis is suitable for the binary outcome variable in this study, as it can isolate the independent effect of the target independent variable by controlling for multiple confounding factors. Single-factor and multi-factor logistic regression analyses were used to examine the associations between PreMetS, MetS, different healthy lifestyles, and multiple comorbidities, with the aim of estimating odds ratios (ORs) and their 95% confidence intervals (CIs). Three models were constructed to progressively adjust for confounding factors: Model 1 was unadjusted; Model 2 was adjusted for age and gender; Model 3 further adjusted for education, marital status, smoking, drinking, sleep, diet, exercise time, total cholesterol (TC), low-density lipoprotein cholesterol (LDL-c), serum uric acid (SUA), creatinine (Cre), and blood urea nitrogen (BUN).

The moderating role of healthy lifestyles was further explored by stratified analyses of healthy lifestyles to explore associations between metabolic status and number of comorbidities, and stratified analyses of metabolic status to explore associations between healthy lifestyles and multiple comorbidities.

The combined concepts of metabolism and healthy lifestyle were explored in relation to the risk of multiple comorbidities by combining three lifestyles (3, 4, 5–6), defined as healthy lifestyles, with normal metabolism and healthy lifestyle as the reference group.

The test level was α = 0.05 (two-sided) and *p* < 0.05 was statistically significant.

## 3 Results

### 3.1 Baseline information

Of the 8,937 subjects included, 3,888 were female (43.5%) with a mean age of 57.82 ± 14.16 years. All participants were divided into three groups, of which 1860 were categorized as normal, 4,520 as PreMetS and 2,557 as MetS. Compared to the metabolically normal group, the groups with PreMetS and MetS had significantly higher (*p* < 0.001) proportions of unhealthy lifestyles (unfavorable and moderate) and multiple comorbidities (Table 1) and were more female, of advanced age, with lower education, and with higher (*p* < 0.001) SBP, DBP, TC, TG, LDL-C, Cre, BUN. HDL-C was lower (*p* < 0.001) (Table 2).

**Table 1:** Comparison of baseline characteristics among study subjects.

Variants	Total (*n* = 8,937)	Normal (*n* = 1,860)	PreMetS (*n* = 4,520)	MetS (*n* = 2,557)	*H*	*P*
Complications					447.294	<0.001
Two/more	1,470 (16.4)	191 (10.3)	738 (16.3)	541 (21.2)		
No/one	7,467 (83.6)	1,669 (89.7)	3,782 (83.7)	2,016 (78.8)		
Healthy lifestyle						<0.001
Unfavorable	2,259 (25.3)	269 (14.5)	1,092 (24.2)	898 (35.1)		
Moderate	3,017 (33.8)	562 (30.2)	1,528 (33.8)	927 (36.3)	92.993	
Favorable	2,339 (26.2)	580 (31.2)	1,213 (26.8)	546 (21.4)		
Extremely favorable	1,322 (14.8)	449 (24.1)	687 (15.2)	186 (7.3)		
Gender					15.950	<0.001
Male	3,888 (43.5)	800 (43.0)	2,052 (45.4)	1,036 (40.5)		
Female	5,047 (56.5)	1,059 (57.0)	2,468 (54.6)	1,520 (59.5)		
Age (year)	57.82 ± 14.16	51.14 ± 14.97	58.31 ± 13.92	61.79 ± 12.11	333.420*	<0.001
Educational level						<0.001
High school and above	2,414 (27.0)	741 (39.9)	1,173 (26)	500 (19.6)		
Below high school	6,518 (73.0)	1,117 (60.1)	3,346 (74)	2,055 (80.4)		
Marriage status						0.036
Married/cohabiting	7,690 (86.1)	1,578 (85.0)	3,934 (87.1)	2,178 (85.3)		
Divorced/widowed/separated	1,238 (13.9)	279 (15.0)	585 (12.9)	374 (14.7)		
Residence					34.486	<0.001
Urban	5,412 (60.6)	1,226 (65.9)	2,723 (60.2)	1,463 (57.2)		
Rural	3,525 (39.4)	634 (34.1)	1,797 (39.8)	1,094 (42.8)		
Smoking					8.499	0.014
No	7,028 (78.6)	1,508 (81.1)	3,533 (78.2)	1,987 (77.7)		
Smoking/quitting	1,909 (21.4)	352 (18.9)	987 (21.8)	570 (22.3)		
Drinking					6.900	0.032
No/moderate	8,560 (95.8)	1,799 (96.7)	4,329 (95.8)	2,432 (95.1)		
Alcoholic	377 (4.2)	61 (3.3)	191 (4.2)	125 (4.9)		
BMI					1098.929	<0.001
Normal	4,540 (50.8)	1,378 (74.1)	2,510 (55.5)	652 (25.5)		
Underweight/overweight/obesity	4,397 (49.2)	482 (25.9)	2,010 (44.5)	1,905 (74.5)		
Exercise situation					3.646	0.162
Medium/high	1,759 (19.7)	394 (21.2)	863 (19.1)	502 (19.6)		
Lack	7,178 (80.3)	1,466 (78.8)	3,657 (80.9)	2,055 (80.4)		
Sleep duration (h/d)					47.705	<0.001
7–8	3,906 (43.7)	928 (49.9)	1,969 (43.6)	1,009 (39.5)		
<7 or >9	5,031 (56.3)	932 (50.1)	2,551 (56.4)	1,548 (60.5)		
Diet					44.926	<0.001
Health	3,498 (39.1)	846 (45.5)	1,739 (38.5)	913 (35.7)		
Under/over nutrition	5,439 (60.9)	1,014 (54.5)	2,781 (61.5)	1,644 (64.3)		

*Indicates the F-statistic of the ANOVA.

**Table 2:** Comparison of laboratory test results among study subjects, ( $\bar{x}$ *± s*) or M (P25, P75).

Variants	Total (*n* = 8,937)	Normal (*n* = 1,860)	PreMetS (*n* = 4,520)	MetS (*n* = 2,557)	*F*	*P*
BMI (kg/m^2^)	23.96 ± 3.92	22.08 ± 5.28	23.57 ± 2.93	26.02 ± 3.36	677.459	<0.001
WC (cm)	83.06 ± 11.53	76.5 ± 6.64	81.97 ± 11.56	89.75 ± 10.88	902.559	<0.001
SBP (mmHg)	128.87 ± 18.55	114.85 ± 9.68	128.41 ± 17.53	139.89 ± 18.09	1261.416	<0.001
DBP (mmHg)	79.65 ± 10.26	73.06 ± 6.44	79.43 ± 10.23	84.81 ± 9.72	840.908	<0.001
FBG (mmol/L)	5.64 ± 2.08	4.81 ± 0.47	5.45 ± 1.73	6.59 ± 2.86	481.745	<0.001
OGTT-2 h (mmol/L)	7.48 ± 3.21	6.27 ± 1.70	7.28 ± 3.17	9.06 ± 3.77	393.454	<0.001
TC (mmol/L)	5.08 ± 1.37	4.80 ± 0.93	5.08 ± 1.33	5.30 ± 1.66	70.999	<0.001
TG (mmol/L)	1.3 (0.9, 1.9)	0.9 (0.7, 1.2)	1.2 (0.9, 1.6)	2 (1.6, 2.8)	2769.513*	<0.001
HDL-C (mmol/L)	1.36 ± 0.43	1.56 ± 0.37	1.38 ± 0.38	1.17 ± 0.47	489.322	<0.001
LDL-C (mmol/L)	2.94 ± 1.05	2.68 ± 0.78	2.96 ± 0.87	3.11 ± 1.41	91.455	<0.001
SUA (mmol/L)	337.65 ± 96.65	304.2 ± 80.60	334.61 ± 95.24	367.34 ± 100.93	247.142	<0.001
Cre (mmol/L)	69.35 ± 20.52	66.73 ± 18.49	69.78 ± 21.85	70.5 ± 19.31	20.293	<0.001
BUN (mmol/L)	5 (4.1, 6.1)	4.8 (3.9, 5.8)	5 (4.1, 6.2)	5.2 (4.2, 6.2)	71.217	<0.001

BMI, Body mass index; SBP, Systolic Blood Pressure; DBP, Diastolic Blood Pressure; FBG, Fasting Blood Glucose; TC, Total Cholesterol; HDL-C, High-Density Lipoprotein Cholesterol; LDL-C, Low-Density Lipoprotein Cholesterol; BUN, Blood Urea Nitrogen.

*Indicates the statistic of the nonparametric rank-sum test.

### 3.2 Association of MetS and its components with multiple comorbidities

After adjusting for covariates in Models 2 and 3, PreMetS and MetS were associated with a significantly higher risk of multiple comorbidities compared to the normal group (*p* < 0.001), with a trend toward an increased risk of multiple comorbidities as the metabolic disorder component increased (*p* < 0.001) (Table 3).

**Table 3:** Association between metabolic syndrome and its components and multiple comorbidities.

Mets and components	OR (95% CI)^a^	*P*	OR (95% CI)^b^	*P*	OR (95% CI)^c^	*P*
Normal	Reference	NA	Reference	NA	Reference	NA
PreMetS	1.70 (1.44–2.02)	<0.001	1.48 (1.25–1.76)	<0.001	1.38 (1.16–1.64)	<0.001
MetS	2.34 (1.96–2.80)	<0.001	1.88 (1.57–2.25)	<0.001	1.61 (1.32–1.97)	<0.001
*P*_trend_	NA	<0.001	NA	<0.001	NA	<0.001

a Model 1: Uncorrected.

b Model 2: Corrected for gender, age.

c Model 3: Model 2 + education, marital status, smoking, drinking, sleep, diet, exercise time, TC, LDL-c, SUA, Cre, and BUN.

### 3.3 Healthy lifestyle and its components associated with multiple comorbidities

After adjusting for covariates in Models 2 and 3, components of a healthy lifestyle, including never smoking (*p* = 0.011), 7-8 h sleeping time (*p* < 0.001), and normal BMI (*p* < 0.001) were associated with a reduced risk of multiple comorbidities. However, no association was found between non-alcohol consumption, moderate alcohol consumption and moderate-intensity exercise with multiple comorbidities (*p* > 0.05). Adherence to a healthy lifestyle (favorable and extremely favorable) was associated with a reduced risk of multiple comorbidities (*p* < 0.001), with a tendency for the risk of multiple comorbidities to decrease as the number of healthy lifestyles increased (*p* < 0.001) (Table 4).

**Table 4:** Association between healthy lifestyle and its components and multiple comorbidities.

Healthy lifestyle	OR (95% CI)^a^	*P*	OR (95% CI)^b^	*P*	OR (95% CI)^c^	*P*
No smoking	0.91 (0.80–1.04)	0.164	0.78 (0.66–0.92)	0.004	0.80 (0.68–0.95)	0.011
No/moderate drinking	1.30 (0.96–1.76)	0.089	1.22 (0.90–1.66)	0.207	1.22 (0.90–1.67)	0.204
Medium/high intensity exercise	0.76 (0.65–0.88)	<0.001	0.82 (0.71–0.96)	0.012	0.87 (0.74–1.01)	0.065
Healthy diet	0.85 (0.76–0.96)	0.007	0.90 (0.80–1.01)	0.066	0.95 (0.84–1.07)	0.369
7–8 h sleeping time	0.60 (0.54–0.68)	<0.001	0.67 (0.60–0.76)	<0.001	0.70 (0.62–0.79)	<0.001
Normal BMI	0.67 (0.60–0.75)	<0.001	0.68 (0.61–0.77)	<0.001	0.70 (0.62–0.78)	<0.001
Lifestyle score						
Unfavorable	Reference	NA	Reference	NA	Reference	NA
Moderate	0.91 (0.80–1.05)	0.199	0.88 (0.77–1.02)	0.08	0.93 (0.80–1.07)	0.297
Favorable	0.61 (0.53–0.72)	<0.001	0.62 (0.53–0.73)	<0.001	0.69 (0.59–0.82)	<0.001
Extremely favorable	0.42 (0.34–0.52)	<0.001	0.45 (0.36–0.56)	<0.001	0.54 (0.43–0.68)	<0.001
*P*_trend_	NA	<0.001	NA	<0.001	NA	<0.001

a Model 1: Uncorrected.

b Model 2: Corrected for gender, age.

c Model 3: Model 2 + education, marital status, smoking, drinking, sleep, diet, exercise time, TC, LDL-c, SUA, Cre, and BUN.

### 3.4 Effect of healthy lifestyle on PreMetS and association of MetS with multiple comorbidities

After stratification according to healthy lifestyle, PreMetS was not associated with multiple comorbidities in healthy lifestyle (moderate and above) (*p* > 0.05), while MetS remained an associated risk factor for multiple comorbidities (*p* < 0.05) (Table 5).

**Table 5:** Association between PreMetS and MetS and multiple comorbidities stratified by healthy lifestyle.

Healthy lifestyle	MetS	OR (95% CI)^a^	*P*	OR (95% CI)^b^	*P*	OR (95% CI)^c^	*P*
Unfavorable							
	Normal	Reference	NA	Reference	NA	Reference	NA
	PreMetS	1.57 (1.24–1.99)	<0.001	1.44 (1.13–1.82)	0.003	1.42 (1.12–1.81)	0.004
	MetS	1.74 (1.36–2.21)	<0.001	1.48 (1.16–1.89)	0.002	1.47 (1.14–1.90)	0.003
Moderate							
	Normal	Reference	NA	Reference	NA	Reference	NA
	PreMetS	1.40 (1.03–1.88)	0.029	1.25 (0.92–1.69)	0.154	1.23 (0.90–1.67)	0.188
	MetS	2.19 (1.59–3.02)	<0.001	1.84 (1.33–2.56)	<0.001	1.84 (1.31–2.58)	<0.001
Favorable							
	Normal	Reference	NA	Reference	NA	Reference	NA
	PreMetS	1.70 (1.05–2.76)	0.030	1.31 (0.80–2.15)	0.287	1.24 (0.75–2.06)	0.401
	MetS	3.67 (2.13–6.32)	<0.001	2.43 (1.38–4.28)	0.002	2.19 (1.21–3.96)	0.009
Extremely favorable							
	Normal	Reference	NA	Reference	NA	Reference	NA
	PreMetS	2.39 (0.60–9.44)	0.214	1.79 (0.44–7.33)	0.420	1.54 (0.36–6.61)	0.560
	MetS	9.92 (2.33–42.24)	0.002	7.32 (1.65–32.36)	0.009	6.27 (1.35–29.08)	0.019

a Model 1: Uncorrected.

b Model 2: Corrected for gender, age.

c Model 3: Model 2 + education, marital status, smoking, drinking, sleep, diet, exercise time, TC, LDL-c, SUA, Cre, and BUN.

### 3.5 Integrated analysis of the association of PreMetS, MetS and unhealthy lifestyle with multiple comorbidities

Integration of three degrees of lifestyle (3, 4, 5–6) defined as healthy lifestyles, and normal metabolism and healthy lifestyles were used as a reference group to explore their association with the risk of multiple comorbidities. Compared to normal metabolic subjects with healthy lifestyles, PreMetS subjects with unfavorable lifestyles were associated with increased risk of multiple comorbidities (*p* = 0.002), whereas PreMetS subjects with healthy lifestyles were not associated with increased risk of multiple comorbidities (*p* > 0.05) (Table 6).

**Table 6:** Association between PreMetS and MetS and unhealthy lifestyle and multiple comorbidities.

Variants	OR (95% CI)^a^	*P*	OR (95% CI)^b^	*P*	OR (95% CI)^c^	*P*
Normal + healthy lifestyle	Reference	NA	Reference	NA	Reference	NA
Normal+ Unfavorable lifestyles	2.51 (1.60–3.93)	<0.001	2.23 (1.42–3.50)	<0.001	2.05 (1.30–3.23)	0.002
PreMetS + healthy lifestyle	1.90 (1.16–3.11)	<0.001	1.59 (0.97–2.60)	0.068	1.52 (0.93–2.50)	0.097
PreMetS + unfavorable lifestyles	3.95 (2.58–6.06)	<0.001	3.14 (2.04–4.83)	<0.001	2.83 (1.83–4.37)	<0.001
MetS + healthy lifestyle	5.07 (2.94–8.76)	<0.001	3.82 (2.20–6.62)	<0.001	3.65 (2.10–6.36)	<0.001
MetS + unfavorable lifestyles	4.96 (3.23–7.64)	<0.001	3.66 (2.37–5.65)	<0.001	3.28 (2.11–5.10)	<0.001

a Model 1: Uncorrected.

b Model 2: Corrected for gender, age.

c Model 3: Model 2 + education, marital status, smoking, drinking, sleep, diet, exercise time, TC, LDL-c, SUA, Cre, and BUN.

### 3.6 Sensitivity analyses

**Figure 1:**
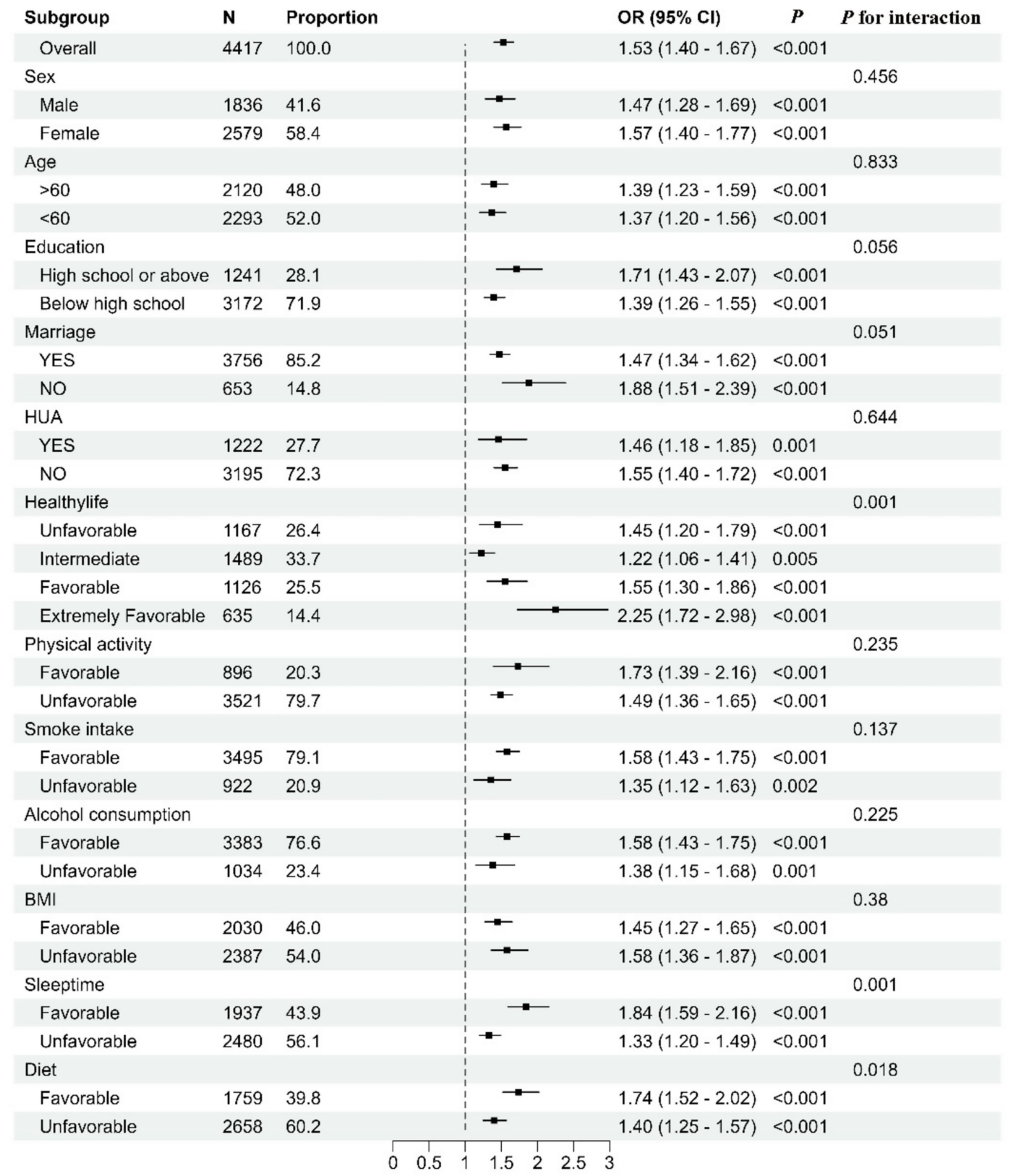
Subgroup analysis.

Subgroup analyses showed consistent and statistically significant associations between MetS and multiple comorbidities in all subgroups, and all showed positive associations. In the MetS stratified analyses, the association between poor lifestyle and multiple comorbidities was shown using healthy lifestyle as a reference. There is an interaction between MetS and poor lifestyle (*p* = 0.001). We also found an interaction between poor diet quality and sleep duration with metabolic status (*p* = 0.018 and 0.001) (Figure 1).

## 4 Discussion

Many epidemiological studies have shown that metabolic disorders are key factors in the development of comorbidities. The results of the present study showed that PreMetS and MetS were positively associated with multiple comorbidities, and this association remained significant even after controlling for confounding variables. In addition, a combination of healthy lifestyle factors was negatively associated with the risk of multiple comorbidities even after controlling for biological mediators such as blood pressure, glucose, and lipids. To our knowledge, this is the first large community-based study to explore the association between metabolic disorders and multiple comorbidities. In this study, participants with more healthy lifestyles had a significantly lower risk of multiple comorbidities, which is consistent with previous studies (34).

The present study shows that both PreMetS and MetS are associated with multiple comorbidities. Sterile inflammation and oxidative stress are important factors that increase the risk of multiple diseases in patients with MetS (36, 37). It has been shown (38) that PreMetS has less aggregation of adverse metabolic components but is also a risk factor for cardiovascular events (39). In addition, participants with 3 or more healthy lifestyle factors had a significantly lower risk of multiple comorbidities compared with metabolically healthy participants. Unlike previous studies, our study population had a higher proportion of PreMetS than MetS. PreMetS has a low risk but high prevalence of multiple comorbidities, and pharmacological treatment of blood pressure, glucose, and lipids is indeed beneficial but costly and potentially adverse relative to lifestyle changes, making the relationship between the two, which can be counteracted by lifestyle, of major public health significance.

We combined lifestyle with PreMetS and MetS to explore joint associations with multiple comorbidities and found that healthier lifestyles offset the risk of multiple comorbidities in the PreMetS group. Lifestyles such as meat dietary patterns (40), smoking (34), alcohol consumption (41), obesity and physical activity (42) were associated with oxidative stress, chronic low-grade inflammation and markers of endothelial function, and co-regulated the physiopathology of comorbidities. Previous studies have shown that adherence to a healthy lifestyle reduces mortality in people with PreMetS and MetS (43). Furthermore, the more healthy lifestyle factors are present, the lower the risk of these diseases (44). Thus, following a healthier lifestyle may lead to favorable changes in inflammatory biomarkers of PreMetS, thus counteracting the negative effects of metabolic disorders. However, this change does not appear to be sufficient to counteract the adverse effects of MetS; instead, the association between MetS and multiple comorbidities was instead strongest in those with the highest lifestyle adherence, possibly because multiple comorbid diagnoses led to this lifestyle change. This leads to the Neiman bias, which reduces the level of exposure to risk factors and thereby weakens the true association between exposure factors and disease. This bias is particularly common in chronic disease research and must be controlled through rigorous design and analytical methods.

With regard to the components of the healthy lifestyle score, nonsmoking, adequate sleep duration, and BMI were associated with a significant reduction in the risk of more than 2 comorbidities, but no beneficial effects of moderate alcohol consumption, moderate-to high-intensity exercise, and diet were found in this study. A large number of previous studies have demonstrated the benefits of exercise on comorbidities (45) and MetS (46), and in the present study this effect still had a downward trend after adjusting for multivariate variables; it is possible that the limited sample size influenced this association between exercise and more than 2 comorbidities. As for alcohol consumption, one study showed that moderate alcohol consumption is associated with a reduced risk of comorbidities in the general population (47), but alcohol consumption may also increase metabolic disorders (48), which may have both positive and negative effects. As for diet, the classical nutritional factors as risk factors for comorbidities are mainly meat, vegetarian food and fruits, but there is a lack of additional information on Chinese dietary patterns for primary prevention of comorbidities, including high salt and low omnivorous food. Previous studies have shown that poor diet contributes to 57.99 and 15.32% of CVD and cancer mortality in Chinese (49), and that Chinese people consume too much sodium while their intake of whole grains is far from optimal. In addition, study participants with 5–6 lifestyles were more strongly negatively associated with more than 2 comorbidities compared to individual healthy lifestyles, suggesting that there may be a joint effect of these lifestyle factors, and that the combined effect of the healthy lifestyle factors on the primary prevention of comorbidities is more pronounced, whereas the individual effect of each factor may be negligible.

In the present study, subgroup analyses and interaction tests showed that the positive association of MetS with more than 2 comorbidities was consistent across subgroups. Being overweight and obese substantially increased the published risk in the MetS population, suggesting a stronger association of MetS with multiple comorbidities in the overweight and obese population. The present study also found that MetS had a stronger positive association with multiple comorbidities in the good lifestyle population. Excessively disturbed metabolic levels in the poor lifestyle population biased the results of the subgroup analyses to some extent, masking the true impact of MetS on the incidence of multiple comorbidities; in other words, the impact of MetS on multiple comorbidities was greater in the population with good lifestyles. Therefore, when formulating health strategies for the prevention and control of Mets and chronic diseases, the PreMetS population should be the core target of primary prevention, and comprehensive lifestyle interventions for this population are more cost-effective. In clinical practice, lifestyle assessments should be incorporated into routine diagnosis and treatment.

## 5 Conclusion

Metabolic profiles and lifestyle factors were independently associated with multiple comorbidities, and a healthy lifestyle counteracted the deleterious effects of PreMetS on the risk of multiple comorbidities in adults in Fuzhou. For this reason, people with PreMetS should adopt a healthy lifestyle to reduce the risk of other diseases.

## 6 Limitations and perspectives

To our knowledge, this is the first study to explore the association between lifestyle, PreMetS and MetS, and risk of multiple comorbidities, and the relative homogeneity of all participants living in one city for a long period of time will reduce confounding. However, there are some potential limitations of this study. First, the participants in this study were all residents of coastal areas in China. The study population may have been unevenly distributed, leading to residual confounding factors. Future studies should expand the sample size and population distribution. Secondly, because the diagnosis of disease in comorbidities was determined by verbal questioning, there is potential for recall bias. Due to the complexity of the project, we only inquired about a limited number of diseases with a high public health burden in Fuzhou City, and it is necessary to further investigate other diseases in future studies. Finally, this study was a single-center observational study design; therefore, causality should be interpreted with caution considering reverse causality and residual or unknown confounding factors. In future research, priority should be given to conducting randomized controlled trials of the “PreMetS lifestyle intervention” to verify the specific effects of healthy diets and exercise and provide more reliable causal evidence for policy-making.

## Data Availability

The raw data supporting the conclusions of this article will be made available by the authors, without undue reservation.
