# Supplementary material for: Moderating effects of a healthy lifestyle on the association of pre-metabolic syndrome with multiple chronic disease comorbidities
Source: Front Public Health. 2025 Aug 6;13:1652015. doi: 10.3389/fpubh.2025.1652015 (PMC12364662; doi:10.3389/fpubh.2025.1652015)
Supplement: Supplementary file 1 [file Table_1.DOCX]

Supply table 1 Comparison of the PreMetS and MetS prevalence rates based on three MetS criteria.

|  | PreMetS | | MetS | |
| --- | --- | --- | --- | --- |
|  | 人数（粗患病率） | 标化患病率 | 人数（粗患病率） | 标化患病率 |
| All(n=8997) |  |  |  |  |
| Revised ATP III | 4470(49.68%) | 48.53% | 2795(31.07%) | 23.59% |
| IDF | 956(10.63%) | 9.77% | 1926(21.41%) | 16.30% |
| JCDCG | 4466(49.64%) | 46.72% | 2687(29.87%) | 22.52% |
| Male(n=3925) |  |  |  |  |
| Revised ATP III | 2053(52.31%) | 49.87% | 1124(28.64%) | 24.47% |
| IDF | 384(9.78%) | 9.77% | 757(19.29%) | 16.78% |
| JCDCG | 1993(50.78%) | 48.05% | 1220(31.08%) | 26.23% |
| Female(n=5072) |  |  |  |  |
| Revised ATP III | 2417(47.65%) | 47.40% | 1671(32.95%) | 23.17% |
| IDF | 572(11.28%) | 9.74% | 1169(23.05%) | 16.11% |
| JCDCG | 2473(48.76%) | 45.56% | 1467(28.92%) | 19.97% |
